# QuEChERS-液相色谱-四极杆-飞行时间质谱法同时筛查干枸杞和桑葚中的农药和真菌毒素

**DOI:** 10.3724/SP.J.1123.2025.06009

**Published:** 2025-12-08

**Authors:** Hang YIN, Yujie XIE, Mengjie SHI, Xingqiang WU, Kaixuan TONG, Qiaoying CHANG, Chunlin FAN, Hui CHEN

**Affiliations:** 1.中国质量检验检测科学研究院，北京 100176; 1. Chinese Academy of Quality and Inspection & Testing，Beijing 100176，China; 2.河北大学化学与材料科学学院，河北 保定 071002; 2. College of Chemistry and Materials Science，Hebei University，Baoding 071002，China

**Keywords:** 药食两用物质, 枸杞, 桑葚, 农药残留, 真菌毒素, 液相色谱-四极杆-飞行时间质谱, QuEChERS, medicinal and edible substances, goji berry, mulberry, pesticide residues, mycotoxins, liquid chromatography-quadrupole-time of flight mass spectrometry （LC-Q-TOF/MS）, QuEChERS

## Abstract

枸杞和桑葚中糖分含量较高且受生长环境影响，易受害虫和病菌侵袭，农药残留和真菌毒素是影响其质量安全的主要因素。基于此，本研究应用改进的QuEChERS方法结合液相色谱-四极杆-飞行时间质谱（LC-Q-TOF/MS）开发了一种简单、高通量、灵敏的分析方法，可实现对枸杞、桑葚两种浆果类药食两用物质中172种农药和11种真菌毒素的同时测定。样品经水化后用5%甲酸乙腈提取，4 g无水硫酸镁、1 g氯化钠进行盐析，经无水硫酸镁、*N*-丙基乙二胺（PSA）、十八烷基键合硅胶（C_18_）、多壁碳纳米管（MWCNTs）净化后，以0.1%甲酸水溶液（含5 mmol/L乙酸铵）和0.1%甲酸甲醇溶液为流动相，通过ZORBAX SB-C_18_（100 mm×2.1 mm，3.5 μm）实现分离。采用电喷雾电离、全离子MS/MS扫描正离子模式进行检测，基质匹配曲线外标法定量分析。实验结果表明，该方法可有效降低基质效应，183种化合物在各自的线性范围内均表现出良好的线性关系，线性相关系数（*R* ^2^） 均大于0.995。该方法的筛查限（SDL）为1~50 μg/kg，定量限（LOQ）为5~50 μg/kg。在1倍、2倍和10倍LOQ添加水平下，183种化合物的回收率范围分别为70.0%~118.5%、70.6%~118.8%和71.2%~119.0%，且相对标准偏差均小于20.0%。枸杞和桑葚的日内精密度分别为0.7%~9.8%和1.0%~17.3%，日间精密度分别为0.8%~9.9%和1.5%~16.0%。将该方法用于15批枸杞和10批桑葚中农药残留和真菌毒素检测，共检出16种化合物（含农药13种、真菌毒素3种），含量为5.61~622.47 μg/kg。对检出率高的烟碱类农药（啶虫脒和吡虫啉）进行了初步风险评估，结果显示，啶虫脒和吡虫啉在枸杞中的慢性膳食摄入风险值（%ADI）分别为0.04%和0.02%，二者在枸杞中的%ADI均低于100%，均在可接受范围内。结果表明，该方法操作简单，通量高，灵敏度好，适用于枸杞和桑葚中多种农药残留和真菌毒素的定性筛查和准确定量。

## Abstract (en)

Goji berries and mulberries are susceptible to pest and pathogen invasion due to their high sugar content and the impact of growth environment. Their quality and safety are primarily influenced by pesticide residues and mycotoxins. This study has developed a simple， high-throughput， and sensitive analytical method for the simultaneous determination of 172 pesticides and 11 mycotoxins in the medicinal and edible substances of goji berries and mulberries using an improved QuEChERS method coupled with liquid chromatography-quadrupole-time of flight mass spectrometry （LC-Q-TOF/MS）. The sample was hydrated with 7 mL of purified water， and 10 mL of 5% formic acid in acetonitrile was added as the extraction solvent， along with 4 g of anhydrous MgSO_4_ and 1 g of NaCl as extraction salts. The extract was purified using 400 mg of anhydrous MgSO_4_， 150 mg of primary secondary amine （PSA）， 100 mg of octadecylsilane （C_18_）， and 5 mg of multi walled carbon nanotubes （MWCNTs）. The supernatant was dried under nitrogen， and the residue was reconstituted in 1.0 mL of a methanol-water solution （3∶2， volume ratio）， homogenized by ultrasonication， and filtered through a 0.22 μm membrane prior to analysis. Separation was performed on a ZORBAX SB-C_18_ column （100 mm×2.1 mm， 3.5 μm） using a mobile phase consisting of 0.1% formic acid in aqueous solution （containing 5 mmol/L ammonium acetate） and 0.1% formic acid in methanol. Detection was carried out using electrospray ionization in positive mode with full ion MS/MS （All Ions MS/MS） scanning. Quantitation was achieved using a matrix-matched external calibration method. The results showed that this method can effectively reduce matrix effects， and 183 compounds exhibited good linearity within their respective ranges， with linear correlation coefficients （*R* ^2^） greater than 0.995. The screening detection limits （SDLs） and limits of quantification （LOQs） for 172 pesticides in goji berries ranged from 1 to 50 μg/kg and 5 to 50 μg/kg， respectively. For mulberries， the SDLs and LOQs ranged from 1 to 20 μg/kg and 5 to 20 μg/kg， respectively. Additionally， the SDLs for 11 mycotoxins in goji berries were between 1 and 20 μg/kg， with corresponding LOQs of 5 to 50 μg/kg. In mulberries， the SDLs ranged from 1 to 10 μg/kg， while the LOQs were between 5 and 20 μg/kg. Overall， the LOQs of 183 compounds in mulberries were less than 20 μg/kg， and the proportions of pesticides and mycotoxins with LOQs less than 20 μg/kg in goji berries were 98.3% and 81.8%， respectively. At the spiked levels of 1， 2， and 10 times LOQs， the recoveries of 183 compounds ranged from 70.0% to 118.5%， 70.6% to 118.8%， and 71.2% to 119.0%， respectively， with relative standard deviations （RSDs） all less than 20.0%. The intra-day precision and inter-day precision of goji berries were 0.7%-9.8% and 1.0%-17.3%， respectively， whereas for mulberries were 0.8%-9.9% and 1.5%-16.0%， respectively. This method was applied to detect pesticides and mycotoxins in 15 batches of goji berries and 10 batches of mulberries， and a total of 16 compounds （including 13 pesticides and 3 mycotoxins） were detected with contents ranging from 5.61 to 622.47 μg/kg. Both detection rate and average content of pesticides and mycotoxins in goji berries were higher than those in mulberries. In addition， a preliminary risk assessment was conducted on the neonicotinoid pesticides with high detection rates （acetamiprid and imidacloprid）. The results showed that the chronic dietary intake risk values （%ADI） of acetamiprid and imidacloprid in goji berries were 0.04% and 0.02%， respectively. Both values are below 100% of the ADI in goji berries and are within acceptable limits. To ensure the safety and quality of goji berry and mulberry products， it is necessary to enhance the management and storage condition control of pesticides and mycotoxins. The results indicated that this method is simple to operate， highly sensitive， and suitable for high-throughput qualitative screening and accurate quantification of multiple pesticide residues and mycotoxins in both goji berries and mulberries. This method can provide reference for high-throughput screening of pesticide residues and mycotoxins in other berry medicinal and edible substances. Furthermore， it can indirectly promote industrial upgrading， facilitate the internationalization of medicinal and edible substances， and achieve a win-win situation regarding health value and economic benefits through technological innovation and the enhancement of standards.

枸杞（*Lycium barbarum* L*.*）和桑葚（*Morus alba* L*.*）作为中国传统药食两用物质，已在2002年被列入《既是食品又是药品的物品名单》之中^［1］^。两者均属于浆果类水果，经干制后成为兼具药用价值与食疗功能的养生佳品。枸杞富含枸杞多糖、*β*-胡萝卜素等活性成分，具有抗衰老、改善视力、提高代谢能力等益处^［2-4］^，一直深受中国及东南亚地区消费者的喜爱，并且随着全球健康意识的不断提高，在西方饮食中也越来越受欢迎^［5］^。桑葚富含多种生物活性物质，其中包括花青素、多糖等，这些营养物质使得桑葚具有降血脂、预防糖尿病、改善心脑血管、保护神经等作用^［6］^。由于其丰富的营养成分和较高的药用价值，近年来市场对桑葚的需求量也在不断增加。

然而，枸杞和桑葚的生长极易受到各种病虫害的影响，导致其产量和质量下降^［7］^。为了提高产量，在枸杞的种植过程中，通常会使用三唑酮、氟硅唑、戊唑醇和苯醚甲环唑等三唑类化学品杀菌剂和杀虫剂用于控制白粉病和炭疽病^［8］^；在桑葚种植过程中，通常会施用噻虫嗪、抗蚜威、吡虫啉等杀虫剂来防治病虫害（主要为菌核病）^［9］^，这使得枸杞和桑葚中可能存在杀菌剂、杀虫剂等农药残留，影响消费者健康。同时，随着枸杞原浆、桑葚汁和桑葚酸奶等衍生食品的流行，原料中的农药残留问题也逐渐引起了消费者的关注。目前，国家标准GB 2763-2021《食品安全国家标准 食品中农药最大残留限量》规定了药用植物、浆果和其他小型类水果中农药的最大残留限量^［10］^，欧盟也制定了枸杞、桑葚中超过400种农药的最大残留限量^［11］^。因此，为保障枸杞和桑葚质量安全，确保其符合国内和国际限量要求，开发枸杞和桑葚中农药高通量检测技术具有重要意义。

新鲜的枸杞和桑葚中水分含量较高（约80%），但为了具有药用效果通常需要经过干燥加工后食用^［12］^。然而，由于受到干燥和储存条件的限制，以及水果中水分活度及含糖量的影响，干果极易受到真菌的污染，进而产生真菌毒素。这类毒素可导致一系列疾病，如癌症、消化道中毒性白血病、免疫和神经系统疾病^［13］^，严重危害人体健康。随着真菌毒素污染日趋严重，不同国家或组织不断建立和完善相关限量标准，例如，欧盟已对果干中黄曲霉毒素（AF）和赭曲霉毒素A（OTA）设立了最大残留限量值。目前报道的针对药食两用物质中真菌毒素的研究多聚焦于毒性较强的AF和OTA等^［14-16］^，而忽略了其他真菌毒素的危害，如枸杞等干制水果的主要潜在入侵病原真菌（交链孢属毒素）^［17，18］^。因此，开发枸杞和桑葚中多种真菌毒素检测技术是保证其质量安全的另一重要方面。

枸杞或桑葚均为较复杂的样品基质，含有多酚、有机酸、色素和糖类等内源性物质，样品前处理技术在分析检测中起着至关重要的作用。目前，已有文献报道采用固相萃取（SPE）^［19］^、固相微萃取（SPME）^［20］^、分散液液微萃取（DLLME）^［21］^和QuEChERS^［22-24］^等前处理方法对枸杞或桑葚中农药或真菌毒素残留进行分析。QuEChERS方法因试剂耗材消耗量少，并且操作简便，节省时间，符合绿色化学的需求，所以被越来越多检测人员所接受。近年来，研究人员对传统QuEChERS方法进行了大量改进，以适应不同的基质种类和目标化合物对于前处理方法的需求^［25］^。目前对药食两用物质中农药残留或真菌毒素的研究相对较少，现有的文献报道大多只局限于单一类别化合物（农药或真菌毒素）的分析测定，难以满足农药和真菌毒素两类污染物的同时检测，并且多数方法仅适配于单一基质，缺乏通用性。例如，Chen等^［22］^建立了超高效液相色谱-串联质谱法测定枸杞中107种农药残留，Xing等^［23］^建立了枸杞样品中5种链格孢属毒素的UPLC-MS/MS方法，田金凤^［24］^建立了超高效液相色谱-串联质谱法测定桑葚中19种农药残留。由上述文献不难看出LC-MS/MS技术已成为食品中农药残留或真菌毒素检测的主要方法，但该技术存在检测通量不够、分辨率低等问题，并且也无法进行溯源分析。液相色谱-四极杆-飞行时间质谱（LC-Q-TOF/MS）结合了高效液相色谱（HPLC）的分离能力和飞行时间质谱（TOF/MS）的高分辨率、精确的质量精度和全谱扫描信息等优势，已广泛应用于复杂样品的分析，如环境分析、食品安全、药物研发和代谢组学研究等^［26，27］^。鉴于LC-Q-TOF/MS在灵敏度、选择性和准确性方面的显著优势，该技术在枸杞、桑葚等药食两用物质的农药残留和真菌毒素筛查方面展现出广阔的应用前景。

本研究通过优化QuEChERS方法中提取溶剂种类及用量、净化剂种类及用量等参数，结合LC-Q-TOF/MS建立了同时测定枸杞和桑葚干中172种农药及11种真菌毒素的分析方法。对筛查限、定量限、准确度和精密度等方法学参数进行验证后，将该方法应用于15批枸杞样品和10批桑葚样品的测定，并对高检出率的农药进行慢性膳食摄入风险评估。该方法实现了药食两用物质中农药残留和真菌毒素的同时检测，提升了检测效率，可以为药食两用物质的质量安全提供技术支撑。

## 1 实验部分

### 1.1 仪器、试剂与材料

Agilent 1290-6550液相色谱-四极杆-飞行时间质谱仪，配有Dual AJS的ESI源（美国安捷伦公司）；Auto EVA 80全自动平行氮吹浓缩仪（中国厦门睿科集团股份有限公司）；TRIO TM-N涡旋搅拌器（日本AS ONE公司）；SR-2DS型水平振荡器（日本TATEC公司）；Allegra X-30R 离心机（美国 Becakmen Coulter 公司）；Milli-Q超纯水机（美国Millipore公司）；PL602-L电子天平（瑞士Mettler-Toledo公司）；超声波清洗器（中国江苏昆山超声仪器有限公司）。

甲酸、乙酸铵（质谱级，美国Agilent公司）；乙腈、甲醇（色谱级，美国Thermo Fisher公司）；甲酸、氯化钠（NaCl）、无水硫酸镁（MgSO_4_）（分析纯，国药集团化学试剂有限公司）；十八烷基硅烷（C_18_）、*N*-丙基乙二胺（PSA）、多壁碳纳米管（MWCNTs）（上海安谱实验科技股份有限公司）；实验用水均为高纯水（经Milli-Q 超纯水器纯化）。

172种农药标准溶液（质量浓度均为1 000 µg/mL）均购于天津阿尔塔科技有限公司；11种真菌毒素标准溶液均购于天津阿尔塔科技有限公司，其中黄曲霉毒素B_1_、B_2_、G_1_、G_2_（分别缩写为AFB_1_、AFB_2_、AFG_1_、AFG_2_）、T-2毒素、OTA、玉米赤霉烯酮，质量浓度均为1 000 µg/mL；白僵菌素（BEA）、恩镰孢菌素B、交链孢酚单甲醚、腾毒素（TEN），质量浓度均为100 µg/mL。

### 1.2 标准溶液的配制

将172种农药和11种真菌毒素准确移取适量体积于10 mL容量瓶中，用甲醇定容至刻度，摇匀后得到10 mg/L的混合标准储备溶液，于4 ℃避光保存。准确移取上述混合标准储备溶液1 mL于10 mL容量瓶中，用甲醇稀释，最终配制成1 mg/L的混合标准工作液，于4 ℃避光保存。

### 1.3 实验条件

#### 1.3.1 样品制备

15批干枸杞和10批干桑葚均购自当地超市。将上述样品置于-20 ℃冰箱放置12 h以上，待样品冻结后，用组织捣碎机加工成粉末状，置于聚乙烯袋中-20 ℃冷冻保存。

#### 1.3.2 样品前处理

准确称取2.0 g（精确至0.01 g）粉末样品于50 mL离心管中，加入7 mL超纯水，水平涡旋3 min后静置20 min。加入10 mL 5%甲酸乙腈溶液，水平涡旋3 min使其充分混匀。加入4 g无水硫酸镁、1 g氯化钠和1颗陶瓷均质子，振荡3 min，以5 000 r/min 离心5 min。取5 mL上清液于15 mL净化管（内含400 mg MgSO_4_、150 mg PSA、100 mg C_18_、5 mg MWCNTs）中，涡旋3 min，5 000 r/min离心5 min。取2 mL上清液于10 mL玻璃试管，40 ℃水浴氮吹至近干后，用1 mL甲醇-水溶液（3∶2， 体积比）复溶，超声后涡旋10 s，经0.22 µm有机滤膜过滤后，供LC-Q-TOF/MS测定。

#### 1.3.3 色谱条件

色谱柱为ZORBAX SB-C_18_柱（100 mm×2.1 mm， 3.5 μm， Agilent， USA），柱温为40 ℃；进样量为5 μL。流动相：A相为 0.1%甲酸水溶液（含5 mmol/L乙酸铵），B相为0.1%甲酸甲醇溶液。梯度洗脱程序：0~3 min， 1%B； 3~6 min， 1%B~30%B； 6~9 min， 30%B~40%B； 9~15 min， 40%B； 15~19 min， 40%B~60%B； 19~23 min， 60%B~90%B； 23~23.01 min， 90%B~1%B； 23.01~27 min， 1%B。流速为0.4 mL/min。

#### 1.3.4 质谱条件

Q-TOF/MS条件：Dual AJS ESI源；扫描方式：正离子全扫描；全扫描范围：*m/z* 50~1 000；毛细管电压：4 000 V；雾化气体：氮气；雾化气压力：0.14 MPa；鞘气温度：375 ℃；鞘气流速：11.0 L/min；干燥气流速：12.0 L/min；干燥气温度：225 ℃；碎裂电压：345 V。All Ions MS/MS模式条件：碰撞能为15 eV和35 eV。

172种农药和11种真菌毒素的详细质谱信息见表1。

**表 1:** 172种农药和11种真菌毒素的质谱信息、筛查限和定量限

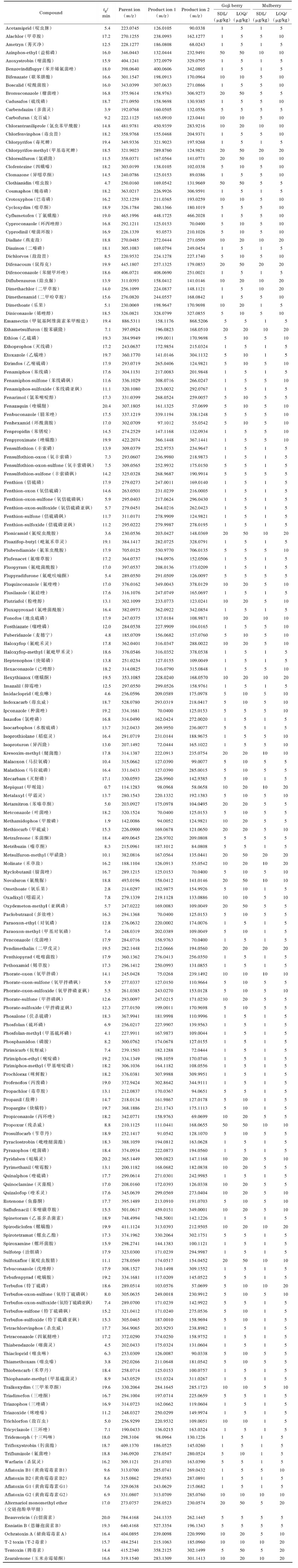

## 2 结果与讨论

### 2.1 前处理条件优化

#### 2.1.1 数据库建立

配制质量浓度为1 000 µg/L的标准溶液，采用LC-Q-TOF/MS在TOF/MS模式下，分别确定每种化合物的保留时间和该化合物的离子化形式（如［M+H］^+^、［M+NH_4_］^+^、［M+Na］^+^），选择响应最高的离子作为加合离子。在数据采集界面依次输入TOF/MS数据库中183种化合物的母离子，将选择的加合离子在Targeted MS/MS模式下，分别采集每种化合物在不同碰撞能量（5~40 eV）下的碎片离子质谱图，并将质谱图导入PCDL软件，形成含有172种农药和11种真菌毒素的精确质量数据库与质谱图库。应用数据库对化合物进行分析，172种农药和11种真菌毒素总离子流图（TIC）见图1。

**图1:**
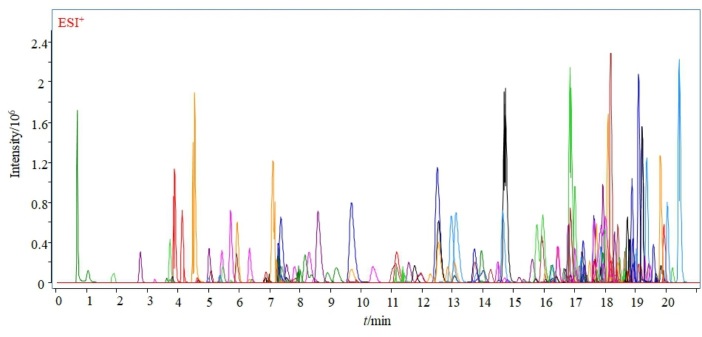
172种农药和11种真菌毒素标准溶液的总离子流色谱图

#### 2.1.2 水化体积的优化

枸杞和桑葚均具有低水、高糖、高酸、黏度大的特点，据报道，含多糖类大分子物质较多的基质在干燥或冻干后样品呈现致密结构，直接加入乙腈提取会使样品凝聚成团，导致化合物无法充分提取，而通过水化可以促进多糖及有机酸的溶解，降低黏性，改善聚团现象^［28］^。因此，本研究在50 µg/kg的添加水平下，考察了不同水化体积（2、5、7 和 9 mL）对化合物回收率的影响。如图2所示，随着水体积的增加，化合物回收率满足要求（70%~120%）的数量呈先升高后下降趋势，当加水量较少时，仅有160种化合物的回收率满足要求，这可能是由于部分化合物的亲水性强，如噻虫嗪、甲基硫环磷、氟喹唑等化合物的log *P*值均小于3，需要加大水化体积以提高亲水性化合物的溶解度，且当加水量不足时，基质中的多糖、蛋白质不能充分溶胀，化合物被包裹在基质中，致使回收率偏低。当加水量达到9 mL时，回收率在70%~120%内的化合物减少，说明水化体积的增加可能会使提取的干扰物增多，从而导致基质效应增强，影响化合物的回收率。当加入7 mL水时，基质分散均匀，满足条件的目标化合物最多，因此，选择水化体积为7 mL。

**图2:**
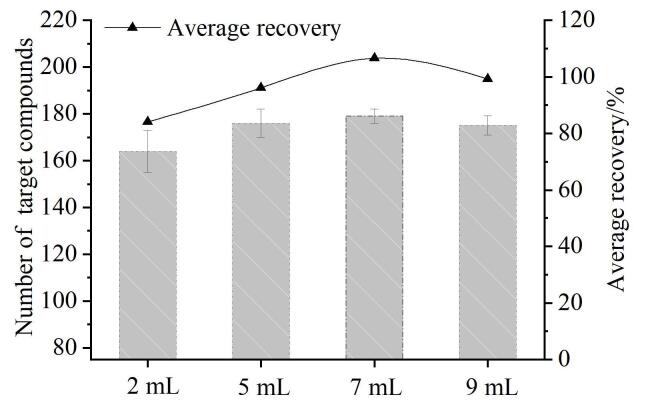
不同水化体积对满足回收率要求的化合物数量及回收率的影响（*n*=3）

#### 2.1.3 提取溶剂的优化

为了降低基质干扰，提高化合物的提取效率，提取溶剂应选择具有与化合物极性接近的试剂。乙腈常用于农产品中农药和真菌毒素的提取^［29］^。因此，本研究在50 µg/kg的添加水平下，比较了乙腈和含不同体积分数甲酸（1%、3%、5%、7%）的乙腈溶液的浸提效率（见图3）。实验发现，与乙腈相比，加入甲酸后部分化合物回收率明显提高，且随着甲酸浓度的升高，p*K*_a_值偏低、酸性条件下以非离子化形式存在的化合物（如多菌灵、亚胺硫磷、AFB_1_）的提取效率呈逐渐上升趋势。当甲酸比例为5%时，化合物提取效率最高；随着甲酸比例升高，部分化合物（如乙螨唑、喹螨醚等）出现回收率降低趋势。因此，选择5%的甲酸乙腈作为下一步分析的最佳提取溶剂。

**图3:**
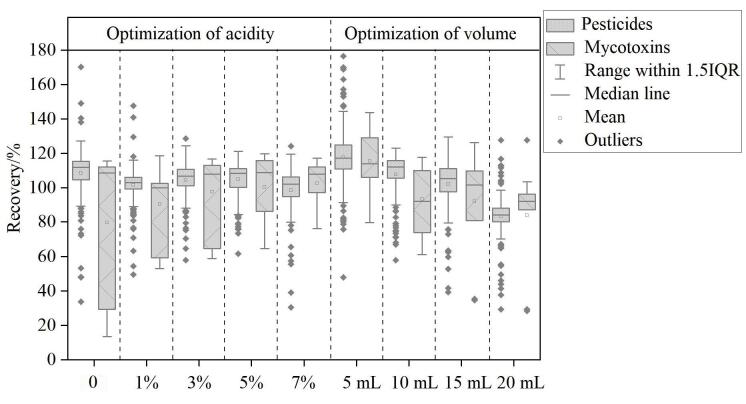
不同提取溶剂（酸度和体积）对化合物回收率的影响

在农药和真菌毒素的提取过程中，溶剂用量对化合物提取效率影响较明显。提取溶剂体积过小，化合物回收率较差，适当的体积会整体改善化合物的回收率，但提取溶剂体积过大时，基质中干扰物的提取效率也随之增加，干扰化合物检测，导致响应降低、回收率下降。因此，本研究考察了5、10、15、20 mL 4种不同提取溶剂体积对化合物提取效率的影响。如图3所示。当提取溶剂体积从5 mL增至10 mL时，对莠灭净、甲基毒死蜱和二甲草胺等65种化合物的回收率具有显著的优化效果，表明提取溶剂用量过低时，化合物提取不充分，导致回收率较差。当溶剂体积为10 mL时 96.7%的化合物回收率在70%~120%范围内。当提取溶剂用量增至15 mL和20 mL时，化合物的检测灵敏度降低，回收率在70%~120%内的化合物占比分别降至94.0%和90.6%。同时，由于提取溶剂体积的增加，造成了试剂浪费。

综合考虑，本研究最终选定10 mL 5%甲酸乙腈作为提取溶剂。

#### 2.1.4 净化剂的优化

在枸杞和桑葚的农药残留和真菌毒素检测中，最佳比例的净化剂是提升检测准确性和可靠性的核心环节。枸杞富含多糖、类胡萝卜素及有机酸等物质，桑葚含大量花青素和多酚类物质，尽管通过盐析和离心沉淀可有效去除提取液中部分内源性大分子干扰物质，但其余干扰物仍会导致基质增强或抑制从而干扰化合物的测定并污染检测系统。因此，需要对提取液进行净化，通过优化净化剂（如PSA、C_18_、GCB或MWCNTs）的组合与配比，可有效去除糖类、色素、有机酸及大分子干扰物，降低干扰物对化合物的影响，提升方法的通用性和重现性。常用的净化剂主要有MgSO_4_、PSA、C_18_等。MgSO_4_通常用于除去提取液中多余的水分，为了避免残留水分对检测结果产生干扰，本研究选择添加400 mg MgSO_4_进行后续优化实验。PSA通过氢键作用能有效去除基质中的糖、有机酸、脂肪酸等大部分极性干扰物，而C_18_可吸附基质中的脂肪等非极性干扰物^［30］^。

本研究以枸杞为样品，在50 μg/kg添加水平下对不同净化剂进行了优化。首先考察了不同用量PSA（50、100、150、200 mg）对化合物的影响。如图4所示，随着PSA用量的增加，化合物回收率在70%~120%内的数量逐渐增多并保持稳定，分别为170、178、181、181。当PSA用量为50 mg时，AFB_1_、AFG_1_的回收率均低于60%。且对于部分农药，如氯磺隆、氧丰索磷砜、噻唑磷、氟酰脲等，PSA用量不足，糖类、有机酸未被充分吸附，无法有效去除干扰物，导致峰形拖尾。当PSA用量增加至150 mg时，不仅降低了干扰物对化合物的影响，同时大部分化合物回收率为70%~120%。进一步增加PSA用量时，化合物的回收率及干扰物的影响没有明显变化，综合各化合物的提取效率和实验成本，选择加入150 mg PSA进行后续实验。

**图4:**
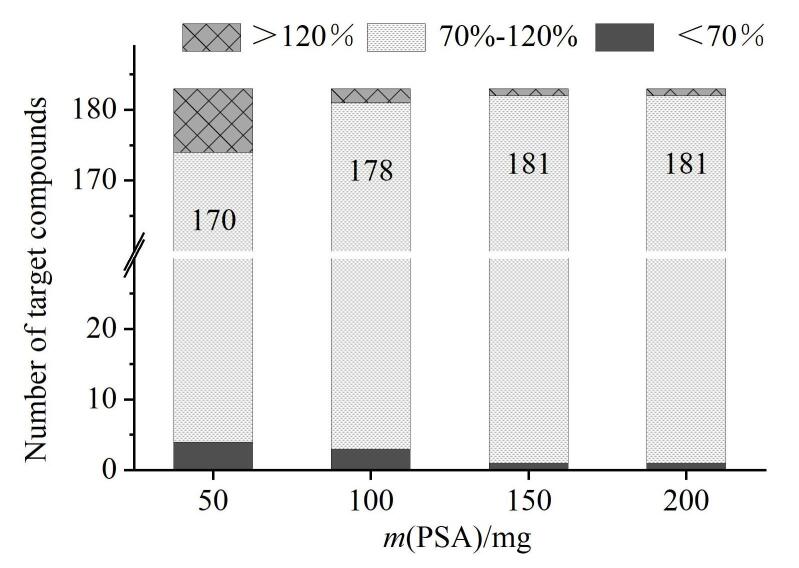
不同用量的PSA对化合物回收率的影响

其次，考察了不同用量C_18_（50、100、150、200 mg）对化合物的影响。结果如图5所示，化合物回收率在70%~120%内的数量呈先上升后降低趋势，分别为177、183、180、175。当C_18_用量为100 mg时，183种化合物均得到满意回收，增加或减少C_18_用量均会降低回收化合物数量。C_18_用量从50 mg增至100 mg后，甲基硫环磷、氟菌唑等农药的回收率增至70%~120%，这表明加入 C_18_能有效吸附基质中的杂质。但随着C_18_用量的继续增加，喹螨醚、氟唑菌酰胺等大部分杂环类化合物的总体回收率呈降低趋势，可能是由于C_18_中的链烷基结构通过疏水作用及范德华力对化合物中的芳香基团产生相互作用力，使得一些非极性和弱极性的农药被吸附，从而影响部分化合物的回收率^［31］^。因此，选择C_18_用量为100 mg。

**图5:**
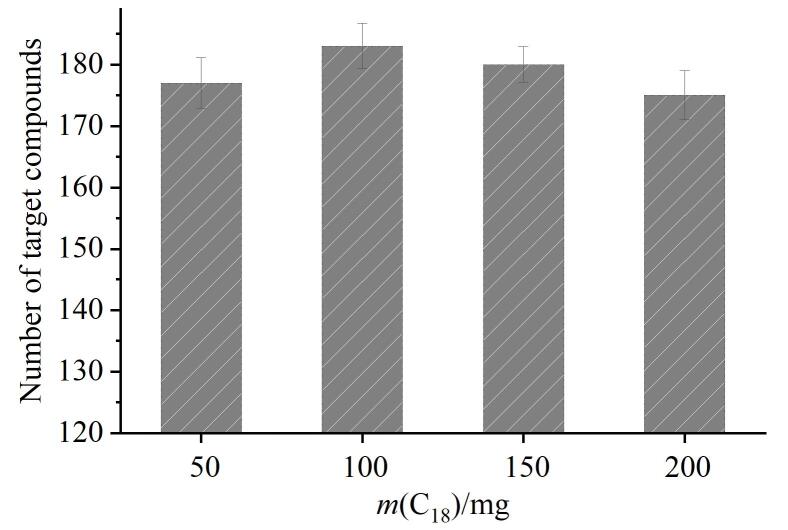
不同用量的C_18_对满足回收率要求的化合物数量的影响（*n*=3）

枸杞样品经上述净化剂提取净化后，其提取液颜色仍较深。因此，需要对提取液进一步净化，以去除色素等干扰物的影响。MWCNTs作为一种新型净化剂，在茶叶、果蔬等多种食品的农药残留检测中显示出了优势^［32-34］^，能有效去除样品中的脂肪酸、色素等干扰物，提升净化效果，但MWCNTs在一定程度上会吸附部分具有平面结构的化合物，影响其回收率^［35，36］^。因此，本研究对MWCNTs用量进行了优化，对比了5、10、15、20 mg MWCNTs对提取液颜色和化合物的影响。结果表明，随着MWCNTs用量的增加，其对色素吸附效果逐渐提升，样液逐渐澄清；但是，随之而来的是部分化合物的回收率呈下降趋势，如麦穗宁、三环唑等。当用量为5 mg时，183种化合物均不会被明显吸附，且提取液颜色明显变浅，当用量超过5 mg时，样液变得澄清透明，但部分化合物回收率逐渐下降。

由于桑葚含有和枸杞类似的活性成分（多糖、多酚等），但桑葚较枸杞色素含量更高，为保证有效去除桑葚中色素干扰，且化合物回收率仍在70%~120%范围内，本研究在50 μg/kg添加水平下，进一步考察了5 mg和10 mg MWCNTs对桑葚中183种化合物的影响。结果表明，当MWCNTs用量为5 mg时，虽然色素去除不显著，但172种农药及11种真菌毒素的回收率呈现满意结果，而加入10 mg时，净化效果明显改善，但部分具有平面结构的农药回收率显著降低，如噻菌灵、咪唑嗪的回收率降低至70%以下，无法满足检测要求（见图6）。综上所述，为使化合物回收率满足要求，本研究选取MWCNTs用量为5 mg。

**图6:**
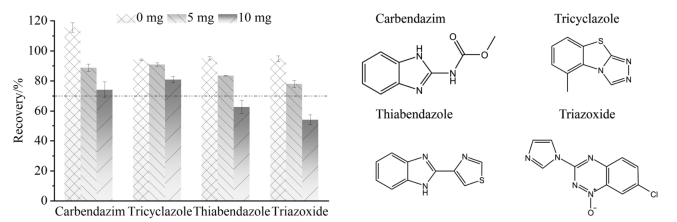
不同用量的MWCNTs对桑葚中部分具有平面结构的化合物回收率的影响（*n*=3）

### 2.2 基质效应评价

基质效应（matrix effect， ME）通常在样品离子化过程中出现，会影响分析结果的准确性和可靠性，不同农药、不同样品基质的基质效应不相同。ME=（基质匹配标准曲线斜率/溶剂标准曲线斜率-1）×100%^［37］^，ME正值为基质增强效应，ME负值为基质抑制效应，其效应强度又分为弱基质效应（|ME|≤20%）、中等基质效应（20%<|ME|≤50%）及强基质效应（|ME|>50%）。

枸杞和桑葚基质成分复杂，基质中组分和共提取物易干扰目标离子，导致离子增强或抑制。本研究通过建立基质匹配标准曲线和溶剂标准曲线计算基质效应（见图7）。结果表明，在枸杞和桑葚基质中分别有67.4%、76.2%的农药和63.6%、63.6%的真菌毒素属于中等和弱基质效应，表明本研究方法对枸杞和桑葚两种浆果类药食同源物质有较强的抗基质干扰能力。

**图7:**
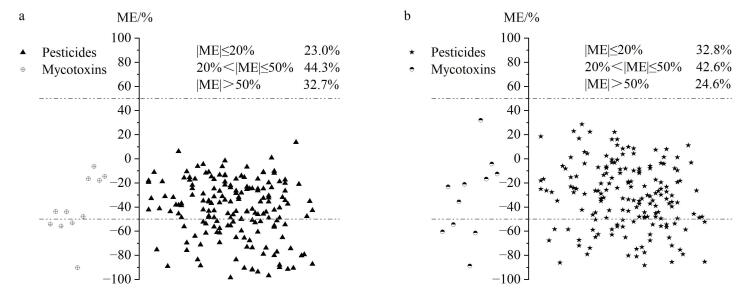
172种农药和11种真菌毒素在（a）枸杞和（b）桑葚中基质效应分布情况

### 2.3 方法学验证

#### 2.3.1 筛查限、定量限和线性

本文对枸杞、桑葚两种基质分别进行了方法学验证，依据SANTE/11312/2021指南文件^［38］^对筛查限和定量限进行验证，具体定义为在一系列添加水平下，每个水平做20组添加实验，检出率达到95%以上的最低浓度即为筛查限（SDL）；定量限（LOQ）为能可靠并准确测定分析物的最低加标浓度（回收率在70%~120%范围内，相对标准偏差（RSD）≤20%。

在最优条件下，枸杞和桑葚的方法学验证结果见表1，17种农药在枸杞中的SDL和LOQ范围分别为1~50 μg/kg 和5~50 μg/kg，在桑葚中的SDL和LOQ范围分别为1~20 μg/kg 和5~20 μg/kg，11种真菌毒素在枸杞中的SDL和LOQ范围分别为1~20 μg/kg 和5~50 μg/kg，在桑葚中的SDL和LOQ范围分别为1~10 μg/kg 和5~20 μg/kg。枸杞中LOQ≤20 μg/kg的农药和真菌毒素占比分别为98.3%和81.8%，桑葚中183种化合物的LOQ均≤20 μg/kg。总体而言，本研究相较于其他方法不仅耗时短、操作简便，还可以有效提高化合物在基质中的灵敏度^［19-21］^。这表明，该方法对两种样品中农药和真菌毒素的检测速度快，灵敏度高，可满足日常筛查需求。向枸杞和桑葚空白基质中添加一系列混合标准工作液，通过校准曲线计算线性相关系数（*R* ^2^）考察回归线的可靠性和准确性。结果显示（见附表，www.chrom-China.com），在线性范围内所有化合物的*R* ^2^为0.995 0~0.999 9，表明各目标物的线性关系良好。

#### 2.3.2 准确度与精密度

为评价方法的准确度与精密度，取空白基质，在1倍LOQ、2倍LOQ和10倍LOQ 3个添加水平进行添加回收试验，每个水平重复6次，计算各添加水平的平均回收率和RSD（见附表）。结果表明，枸杞中3个添加水平的平均回收率分别为70.0%~118.5%、70.6%~118.8%、71.2%~118.5%，RSD范围为0.5%~15.4%。桑葚中3个添加水平的平均回收率分别为71.4%~118.4%、73.2%~118.3%、73.7%~119.0%，RSD范围为0.5%~14.4%。通过在一天内和连续3天内重复分析6次来验证该方法精密度，以RSD表示的枸杞和桑葚中日内精密度均低于10.0%，日间精密度分别为1.0%~17.3%和1.5%~16.0%。表明所建立方法具有良好的准确度和精密度，可同时满足枸杞和桑葚中农药残留和真菌毒素的检测要求。

#### 2.3.3 实际样品检测

将本研究建立的方法用于25份市售样品（其中枸杞15批次、桑葚干10批次）的农药残留和真菌毒素的筛查和定量检测。结果如表2所示，25批次样品中检出13种农药和3种真菌毒素，枸杞和桑葚检出率分别为60%和20%，其中枸杞样品问题发现率较高，15批枸杞样品中共筛查出11种农药及2种真菌毒素，其中农药检出率最高的为新烟碱类杀虫剂（包括啶虫脒和吡虫啉），分别占40.0%和26.7%，这可能与在枸杞种植过程中的广谱杀虫剂使用有关。其余9种农药除螺螨酯、噻虫嗪和炔螨特外均为杀菌剂，这可能与在枸杞种植过程中防治病菌感染有关。本次检出的真菌毒素以AFB_2_为主（检出率33.3%）。值得注意的是，虽然我国尚未制定枸杞中该毒素的最大残留限量标准，但检出样本中有两批次AFB_2_含量已超出欧盟规定的10 μg/kg限量标准。而TEN在4批次枸杞样品中检出，检出频率（26.7%）和检出含量（54.75~180.07 μg/kg）均值得关注。此次检测的10批次桑葚样品中，共检出2种农药（敌敌畏和敌百虫）和1种真菌毒素（BEA），其中2种农药残留含量均低于GB 2763-2021规定的最大残留限量值，相对较为安全，而BEA的检出率为20%，检出含量为5.61~148.98 μg/kg，但该真菌毒素均未在相关法律法规中规定最大残留限量。典型样品的色谱图见图8。上述检测结果表明，在枸杞和桑葚种植过程中可能存在不规范使用农药和不正确干燥或贮藏引起真菌毒素污染的情况，其潜在毒性需引起重视。

**表 2:** 15份枸杞和10份桑葚样品中农药和真菌毒素的检出情况

Compound	Matrix	Detection rate/%	Content/（µg/kg）	MRL^*^/（mg/kg）
Acetamiprid	goji berry	40.0	13.44-581.58	2
Carbendazim	goji berry	20.0	67.95-622.47	1
Difenoconazole	goji berry	13.3	14.47-21.64	/
Imidacloprid	goji berry	26.7	28.01-139.95	1
Paclobutrazol	goji berry	6.7	11.70	/
Propargite	goji berry	13.3	70.61-114.11	10
Pyraclostrobin	goji berry	6.7	72.13	/
Spirodiclofen	goji berry	6.7	21.32	/
Tebuconazole	goji berry	13.3	29.56-182.98	/
Thiamethoxam	goji berry	6.7	186.84	0.5
Thiophanate-methyl	goji berry	20.0	10.78-22.50	/
Aflatoxin B_2_	goji berry	33.3	6.82-31.57	/
Tentoxin	goji berry	26.7	54.75-180.07	/
Dichlorvos	mulberry	10.0	36.79	0.2
Trichlorfon	mulberry	10.0	14.19	0.2
Beauvericin	mulberry	20.0	5.61-148.98	/

* GB 2763-2021.

**图8:**
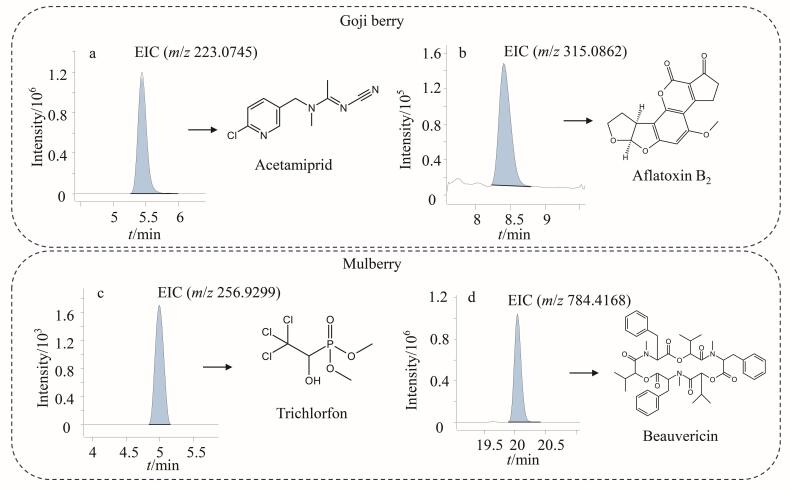
（a）啶虫脒、（b）黄曲霉毒素B_2_、（c）敌百虫和（d）白僵菌素的色谱图

### 2.4 风险评估

实际样品中检出率最高的两种农药（啶虫脒和吡虫啉）均为新烟碱类杀虫剂，这类农药不仅容易被植物吸收，并且具有难去除的特性，从而导致其在农作物中长期残留，最终通过膳食被人体摄入^［39］^。近期越来越多的研究表明，新烟碱类农药及其代谢物可能对人体存在生殖毒性、免疫毒性、肝毒性、肾毒性、遗传毒性、神经毒性等潜在风险^［40］^。因此，有必要基于慢性膳食暴露风险值（%ADI）对检出率高的新烟碱类杀虫剂进行慢性风险评估以考察其对人体存在的潜在危害。

本研究采用欧洲食品安全局（EFSA）的点评估方法，通过计算慢性接触水平，来估算在实际样品中检测到的农药所构成的%ADI。%ADI若小于100%，代表慢性膳食摄入风险在可接受范围内，且%ADI数值越小，慢性摄入风险越低；若%ADI大于100%，则表明慢性摄入风险不可接受。计算公式见式（1）和式（2）：


$$NEDI=\frac{STMR\times F}{bw}$$ （1）

$$\%ADI=\frac{NEDI}{ADI}\times 100\%$$ （2）

式中：NEDI为国家估计的每日摄入量（mg/kg bw）。STMR为检测试验中的中位残留水平（mg/kg）。*F*为某一食品的日平均消费量（kg/d），2020年版《中国药典》将枸杞的摄入量列为0.006~0.012 kg/d，本文计算使用平均值0.009 kg/d作为每日枸杞摄入量^［41］^。成人平均体重（bw）为60 kg。ADI为农药每日可接受的摄入量（mg/kg bw），查得啶虫脒和吡虫啉的ADI值分别为0.07 mg/kg bw和0.06 mg/kg bw。

计算得到啶虫脒和吡虫啉在枸杞中的%ADI值分别为0.04%和0.02%，均远低于100%，表明新烟碱类杀虫剂在枸杞阳性样本中风险可接受。

## 3 结论

本文采用改进的 QuEChERS方法结合LC-Q-TOF/MS检测技术，建立了枸杞和桑葚中172种农药和11种真菌毒素多残留的快速检测方法，在水化体积、提取溶剂的种类和体积以及净化剂的添加量方面进行优化，并进行基质效应评价和方法学验证，结果表明，该方法对枸杞和桑葚中农药和真菌毒素的检测灵敏度高，可满足日常筛查需求，准确度与精密度也呈现良好结果。应用本方法对25批次实际样品进行检测，结果显示，部分样品中存在农药残留及真菌毒素污染问题，对检出率较高的新烟碱类农药进行的慢性膳食风险评估结果显示存在风险较低，但仍有必要加强农药和真菌毒素的使用管理和储存条件控制，以确保枸杞和桑葚产品的安全性和质量。该方法具有简单、快速的特点，不仅可以为其他浆果类药食同源物质中农药残留及真菌毒素的高通量筛查提供参考，也可以间接推动产业升级，促进药食同源物质国际化，通过技术创新与标准完善，实现健康价值与经济效益的双赢。
